# Supplementary material for: Synthesis, Structure and Photochemistry of Dibenzylidenecyclobutanones
Source: Molecules. 2022 Nov 5;27(21):7602. doi: 10.3390/molecules27217602 (PMC9653593; doi:10.3390/molecules27217602)
Supplement: Supplementary file 1 [file molecules-27-07602-s001.zip › Supplementary Materials_revised 2.pdf]

Supplementary Materials Synthesis, Structure, and Photochemistry of Dibenzylidenecyclobutanones

Marina V. Fomina, Alexandra Ya. Freidzon, Lyudmila G. Kuz'mina, Anna A. Moiseeva, Roman. O. Starostin, Nikolai A. Kurchavov, Vyacheslav N. Nuriev, and Sergey P. Gromov*

CONTENT:

I The geometric parameters of molecules **1b-d** and **2**, X-ray diffraction experiments, crystallographic characteristics and structure refinement details, (Tables S1-S5)................................2

II The ^{1}H NMR, ^{13}C NMR, NOESY NMR and HRMS spectra of compounds **1a-f**, NOESY NMR spectrum of compound **1c** (Figures S1-S19)..5

III Fluorescence spectra of compounds **1c-f** (Figure S20) 18

IV Quantum chemical calculations, orbitals involved in the first electron transition of compounds **1b-f** (Figures S21-S25) 19

V Quantum chemical calculations, potential energy profiles of the ground S0 and lowest excited states of compounds **1a-f** (Figures S26-S32) 24

VI Correlations between the calculated frontier orbital energies, ionization potential and electron affinities, and experimental oxidation and reduction potentials (Figures S33-S35)............................28

I X-ray diffraction experiments, crystallographic characteristics and structure refinement details (Tables S1-S5)

Table S1. Selected bond lengths (d, Å) and bond angles (ω,°) in molecules **1b**-**f**.

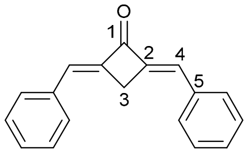

1b-f

Bond/ structure	1b	1c	1d	1e	1f (A)	1f (B)
C1-O1	1.213(2)	1.213(3)	1.219(3)	1.219(4)	1.225(2)	1.223(2)
C1-C2	1.481(2)	1.484(3)	1.480(2)	1.486(5)/ 1.484(5)	1.485(2)/ 1.488(2)	1.486(2)/ 1.488(2)
C2-C3	1.530(2)	1.529(3)	1.530(2)	1.537(5)/ 1.531(5)	1.531(2)/ 1.532(2)	1.534(2)/ 1.526(2)
C2-C4	1.334(2)	1.337(3)	1.335(2)	1.339(5)/ 1.340(5)	1.344(2)/ 1.344(2)	1.339(2)/ 1.340(2)
C4-C5	1.454(2)	1.454(3)	1.451(2)	1.451(5)/ 1.454(5)	1.448(2)/ 1.454(2)	1.453(2)/ 1.455(2)
C2-C1-C2	91.0(2)	90.9(2)	91.1(2)	91.0(3)	90.8(1)	90.8(1)
C1-C2-C3	90.8(1)	90.8(2)	98.8(1)	90.7(3)/ 91.0(3)	91.0(1)/ 90.8(1)	90.7(1)/ 90.9(1)
C2-C3-C2	87.3(2)	87.6(2)	87.3(2)	87.3(2)	87.4(1)	87.6(1)
C1-C2-C4	128.7(1)	129.6(2)	130.1(1)	132.0(3)/ 132.1(3)	131.9(1)/ 132.3(1)	132.5(1)/ 131.8(1)
C3-C2-C4	140.5(1)	139.6(2)	139.0(2)	137.3(3)/ 136.8(3)	136.9(1)/ 136.9(1)	136.8(1)/ 137.2(1)
C2-C4-C5	131.2(1)	130.2(2)	130.6(2)	128.5(3)/ 128.2(3)	128.0(1)/ 128.3(1)	128.5(1)/ 128.4(1)

TableS2. Selected bond lengths (d, Å) and bond angles (ω,°) in molecules *syn,syn*-**2** and *anti,anti*-**2**.

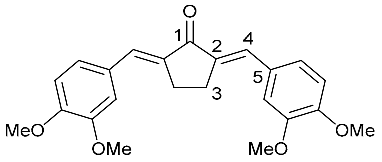

syn,syn-**2**

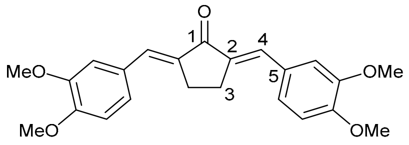

anti,anti-**2**

Bond	d (*syn,syn*-2)	d (*anti,anti*-2)	Angle	ω (*syn,syn*-2)	ω (*anti,anti*-2)
C1-C2	1.484(2)/ 1.483(2)	1.481(3)/ 1.481(3)	C1-C2-C4	120.5(1)/ 120.5(1)	120.7(2)/ 120.4(2)
C2-C4	1.346/ 1.343(2)	1.338(3)/ 1.343(3)	C3-C2-C4	130.4(1)/ 130.8(1)	130.0(2)/ 130.3(2)
C4-C5	1.455/ 1.454(2)	1.459(2)/ 1.456(3)	C2-C4-C5	130.7(1)/ 131.1(1)	131.0(2)/ 131/0(2)

Table S3. Crystallographic parameters and X-ray experiment details for **1b**-**d**.

Dienone	1b	1c	1d

Molecular formula	$C_{20}H_{18}O_3$,	$C_{22}H_{22}O_5$,	$C_{20}H_{18}OS_2$,
Molecular weight, g·mol^{-1}	306.34	366.40	338.46
Temperature, K	296	296	296
Crystal system	Monocl.	Monocl.	Monocl.
Space group	C2/c	C2/c	C2/c
a (Å)	11.9333(5)	17.5713(12)	11.4919(4)
b (Å)	12.6418(5)	13.7149(11)	13.3354(5)
c (Å)	11.0532(5)	8.1128(6)	11.8054(6)
α (°)	90	90	90
β (°)	108.198(2)	103.522(3)	110.904(2)
γ (°)	90	90	90
V (Å^3)	1584.07(12)	1900.9(2)	1690.09(12)
Z	4	4	4
$Å_{calc.,}$ g/sm^3	1.285	1.273	1.330
μ(Mo$K_α$), mm^{-1}	0.086	0.090	
Crystal size	0.54 × 0.46 × 0.42	0.54 × 0.42 × 0.38	0.46 × 0.14 × 0.014
Scan range on 2θ, deg.	4.82 – 52.74	3.8 – 54.62	4.88-53.38
Index range	-14 ≤ h ≤ 14, -15 ≤ k ≤ 15, -13 ≤ l ≤ 13	-22 ≤ h ≤ 22, -17 ≤ k ≤ 17, -10 ≤ l ≤ 10	-13 ≤ h ≤ 14, -16 ≤ k ≤ 14, -14 ≤ l ≤ 14
Number of measured refl.	7871	9410	8466
Number of independent refl. [R(int)]	1617 [Rint = 0.0406, Rsigma = 0.0298]	2110 [Rint = 0.0610, Rsigma = 0.0467]	1747 [Rint = 0.0452, Rsigma = 0.0369]
Number of refl with I >2σ(I)	1323	1577	1377
Number of variables	107	126	107
GOOF	1.052	1.029	1.038
R indices for I > 2σ(I)	R_1 = 0.0423, wR_2= 0.1023	R_1= 0.0520, wR_2 = 0.1302	R_1 = 0.0469, wR_2 = 0.1086
R indices for all refl.	R_1 = 0.0522, wR_2 = 0.1086	R_1 = 0.0710, wR_2 = 0.1427	R_1 = 0.0633, wR_2 = 0.1175
Δρ max/min, eÅ^{-3}	0.12/-0.12	0.17/-0.23	0.21/-0.25

Table S4. Crystallographic parameters and X-ray experiment details for **1e** and **1f**.

Dienone	**1e**	**1f**
Molecular formula	$C_{22}H_{24}N_2O$,	2($C_{26}H_{32}N_2O$),
Molecular weight, g·mol^{-1}	332.43	388.53
Temperature, K	150	150
Crystal system	Monocl.	Monocl.
Space group	P2$_1$/c	P2$_1$/c
a (Å)	7.3571(10)	17.5050(8)
b (Å)	19.831(3)	16.6754(9)
c (Å)	12.3034(17)	15.1879(7)
α (°)	90	90
β (°)	96.130(2)	101.390(2)
γ (°)	90	90
V (Å^3)	1784.8(4)	4346.1(4)
Z	4	8
$ρ_{calc}$, g·sm^{-3}	1.237	1.188
μ(Mo$K_α$), mm^{-1}	0.076	0.072
Crystal size	0.36 × 0.28 × 0.18	0.42 × 0.26 × 0.14
Scan range on 2θ, deg.	5.29-56	3.69-56.0
Index range	9 ≤ h ≤ 9, -26 ≤ k ≤ 26, -	-22 ≤ h ≤ 23, -22 ≤ k ≤ 22, -

	16 ≤ l ≤ 16	17 ≤ l ≤ 20
Number of measured refl.	17935	51909
Number of independent refl. [R(int)]	4225 [Rint = 0.0410, Rsigma = 0.0375]	10460 [Rint = 0.0465, Rsigma = 0.0412]
Number of refl with I >2σ(I)	3321	7598
Number of variables	230	571
R indices for I > 2σ(I)	R_1 = 0.0996, wR_2 =0.3008	R_1 = 0.0572, wR_2 = 0.1306
R indices for all refl.	R_1 = 0.1164, wR_2 = 0.3067	R_1 =0.0882, wR_2 = 0.1413
GOOF	1.133	1.047
Δρ max/min, eÅ^{-3}	0.61/-0.346	0.47/-0.40

Table S5. Crystallographic parameters and X-ray experiment details for **2**.

Dienone	*syn,syn*-**2**	*anti,anti*-**2**
Molecular formula	$C_{23}H_{24}O_5$	$C_{23}H_{24}O_5$
Molecular weight, g·mol^{-1}	380.42	380.42
Temperature, K	150	150
Crystal system	Monocl.	Monocl.
Space group	$P2_1/c$	$P2_1/c$
a (Å)	7.9435(2)	8.7902(4)
b (Å)	7.9435(2)	14.5194(8)
c (Å)	27.5693(6)	15.0411(8)
α (°)	90	90
β (°)	92.4020(10)	95.027(2)
γ (°)	90	90
V, Å^3	1929.22(8)	1912.29(17)
Z	4	4
$\rho_{\chi alc}$, g·sm^{-3}	1.310	1.321
μ(MoK_α), mm^{-1}	0.092	0.092
Crystal size, mm	0.44 × 0.18 × 0.05	0.46 × 0.02 × 0.01
Scan range on 2*θ*, deg.	4.85 to 52.718	4.652 to 52.788
Index range	-9 ≤ h ≤ 9, -10 ≤ k ≤ 10, -34 ≤ l ≤ 34	-10 ≤ h ≤ 10, -18 ≤ k ≤ 18, -18 ≤ l ≤ 18
Number of measured refl.	18751	17719
Number of independent refl. [R(int)]	3909 [Rint = 0.0361, Rsigma = 0.0298]	3899 [Rint = 0.0544, Rsigma = 0.0437]
Number of refl with *I* > 2σ(*I*)	3017	2960
Number of variables	338	273
R indices for *I* > 2σ(*I*)	R_1 = 0.0382, wR_2 = 0.0884	R_1 = 0.0496, wR_2 = 0.1013
R indices for all refl.	R_1 = 0.0546, wR_2 = 0.0963	R_1 = 0.0703, wR_2 = 0.1094
GOOF	1.022	1.043
Residuals, min/max, e/Å^3	0.18/-0.17	0.18/-0.25

II The ^{1}H NMR, ^{13}C NMR, NOESY NMR and HRMS spectra of compounds 1a-f (Figures S1-S19)

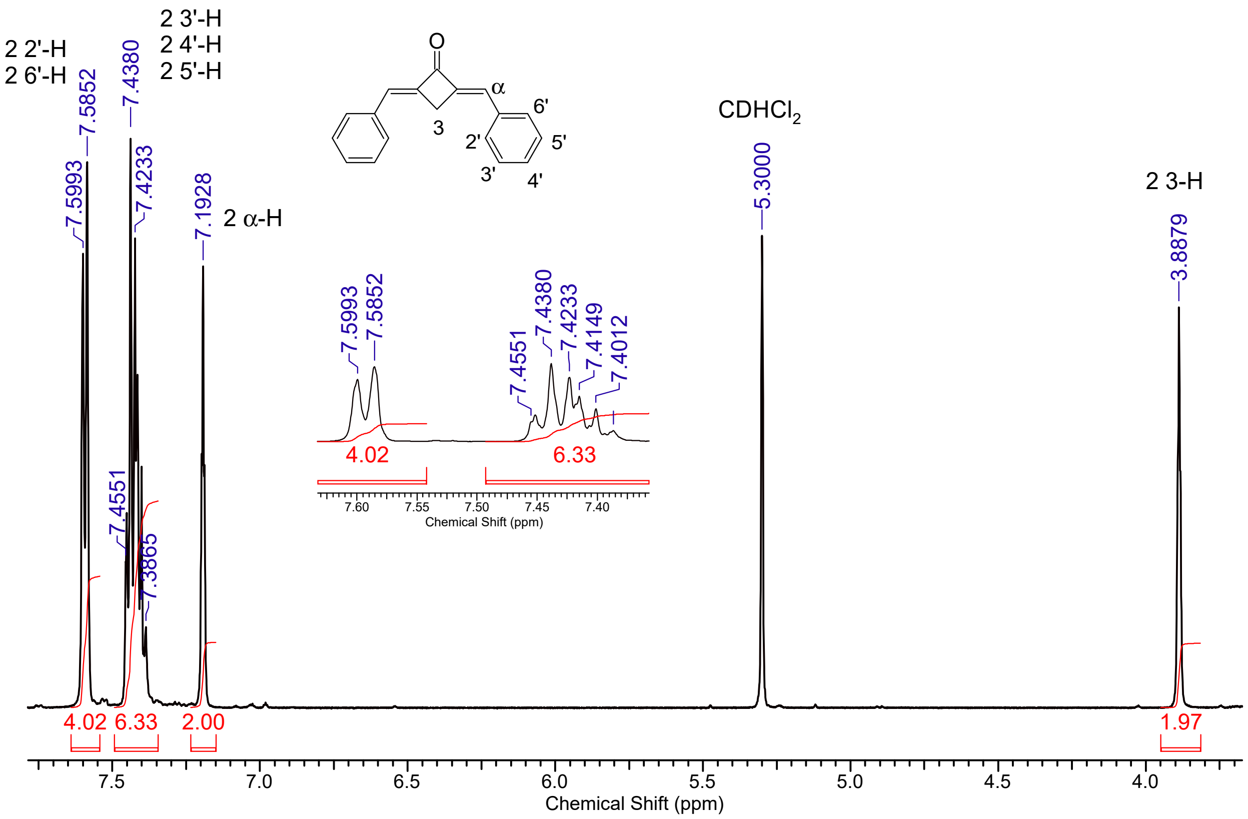

Figure S1. ^{1}H NMR spectrum of compound **1a** in CD_2Cl_2.

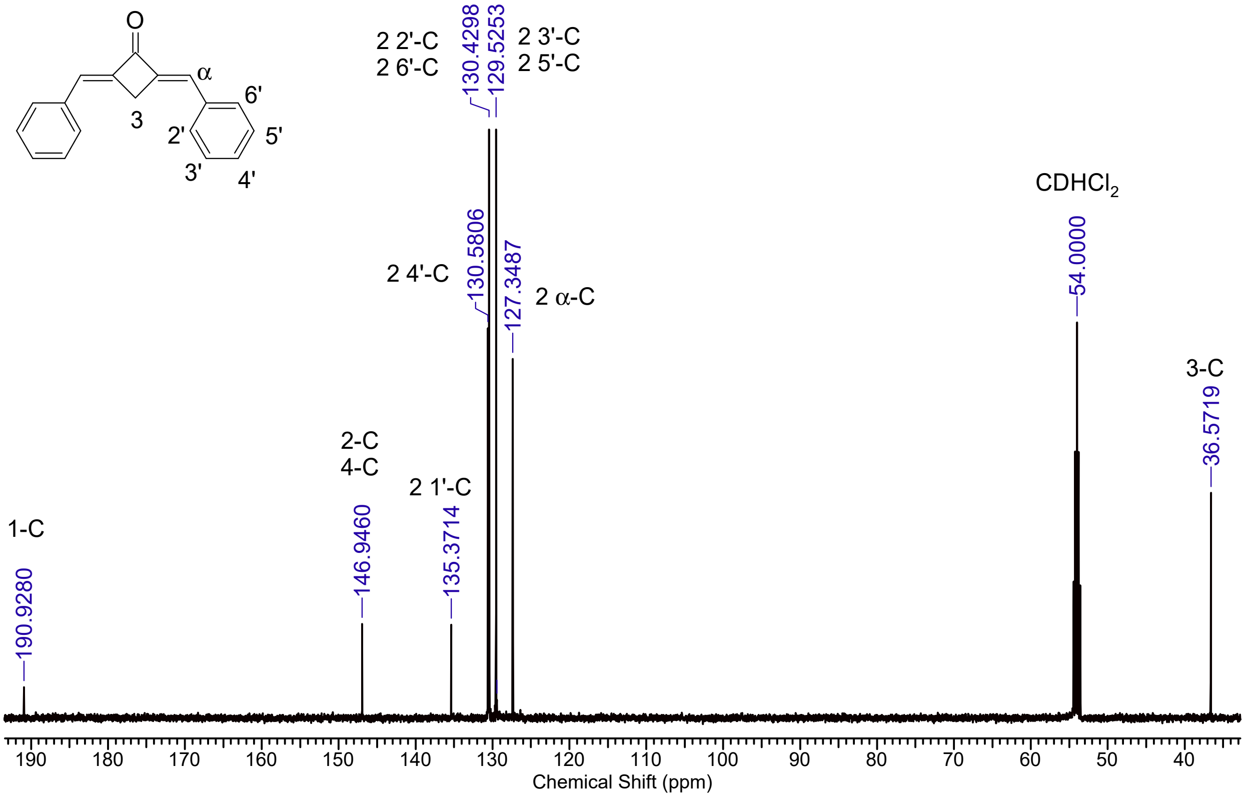

Figure S2. ^{13}C NMR spectrum of compound **1a** in CD_2Cl_2.

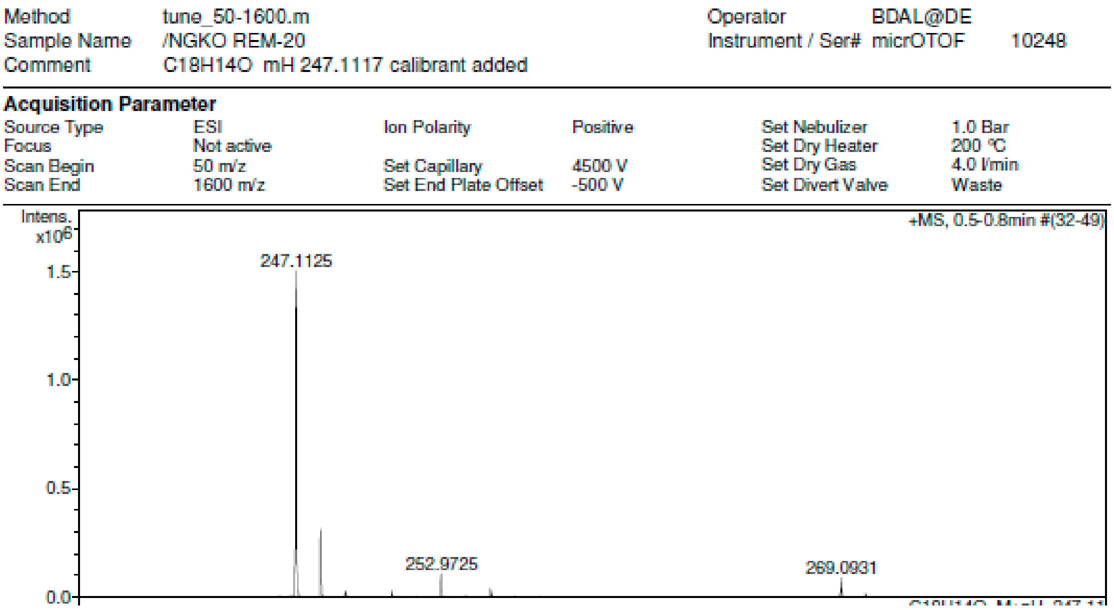

Figure S3. HRMS spectrum of compound **1a**.

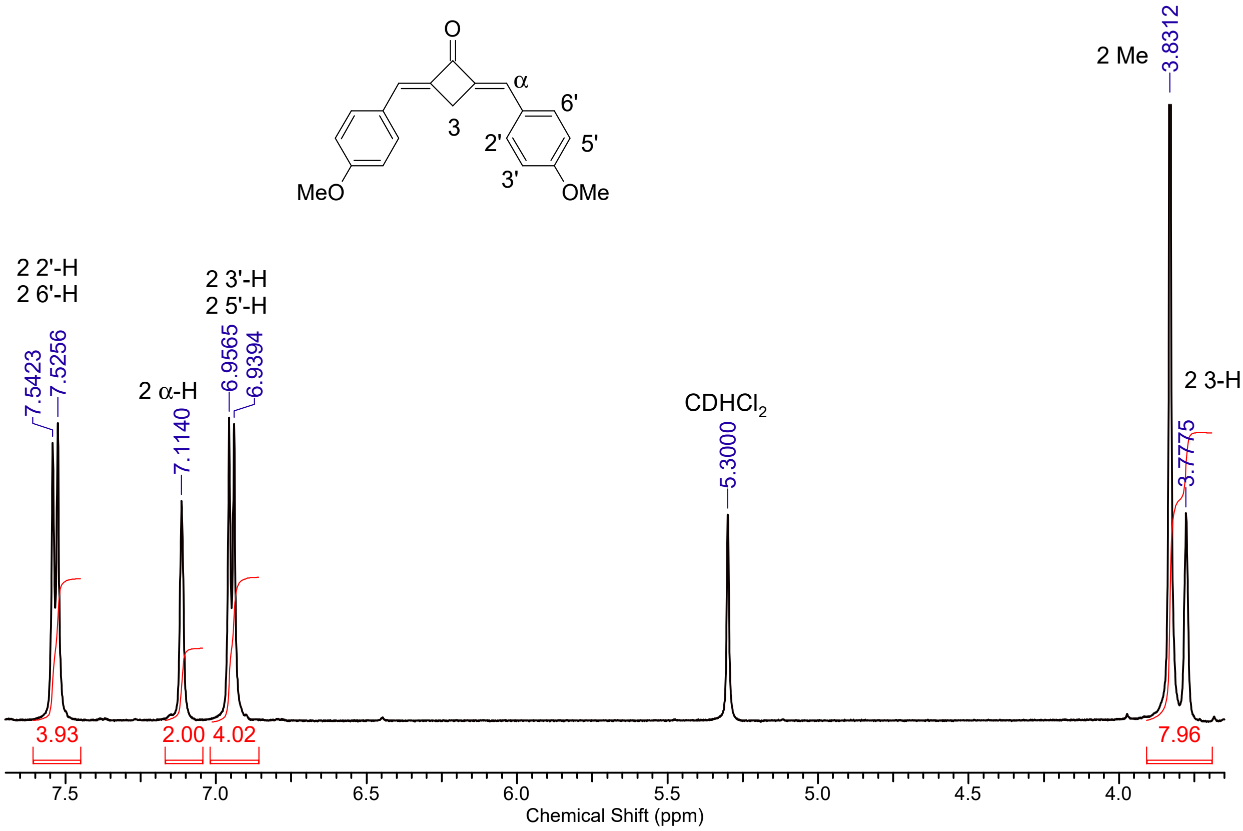

Figure S4. ^{1}H NMR spectrum of compound **1b** in CD_2Cl_2.

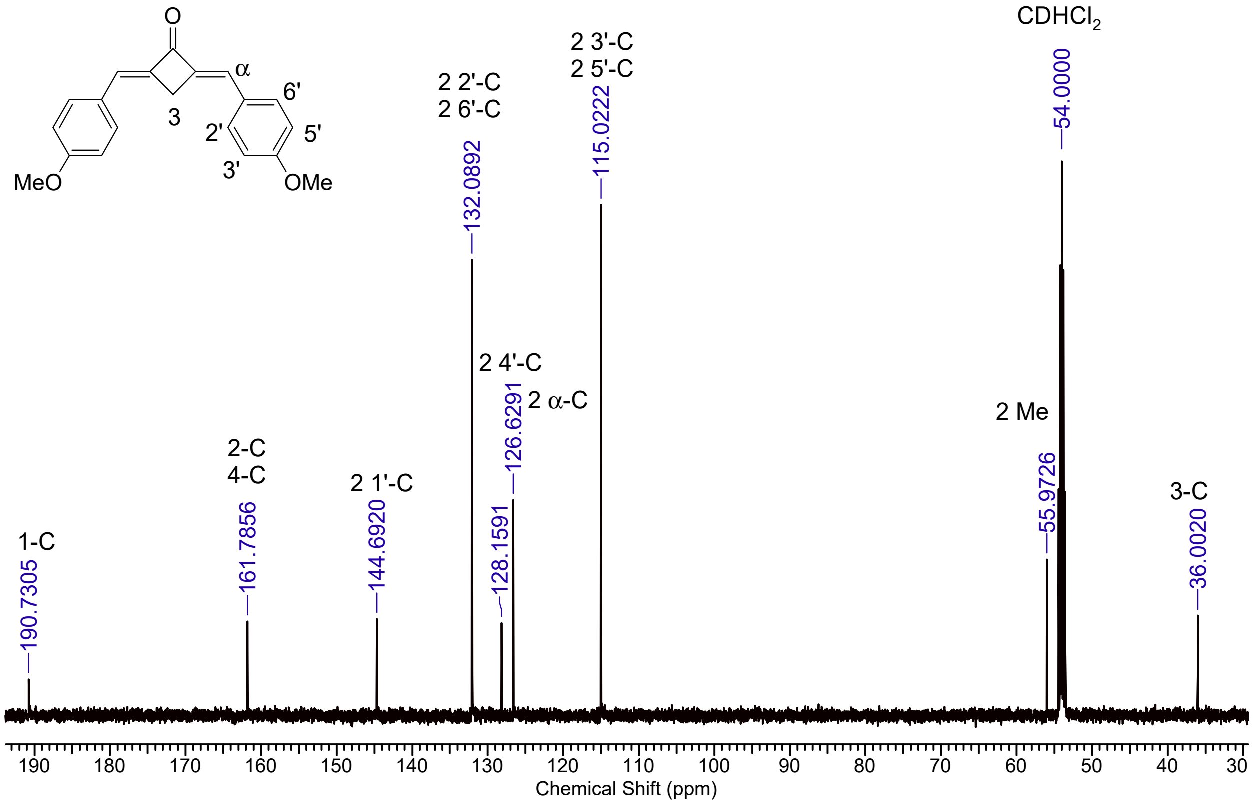

2 3’-OMe

Figure S5. ^{13}C NMR spectrum of compound **1b** in CD_2Cl_2.

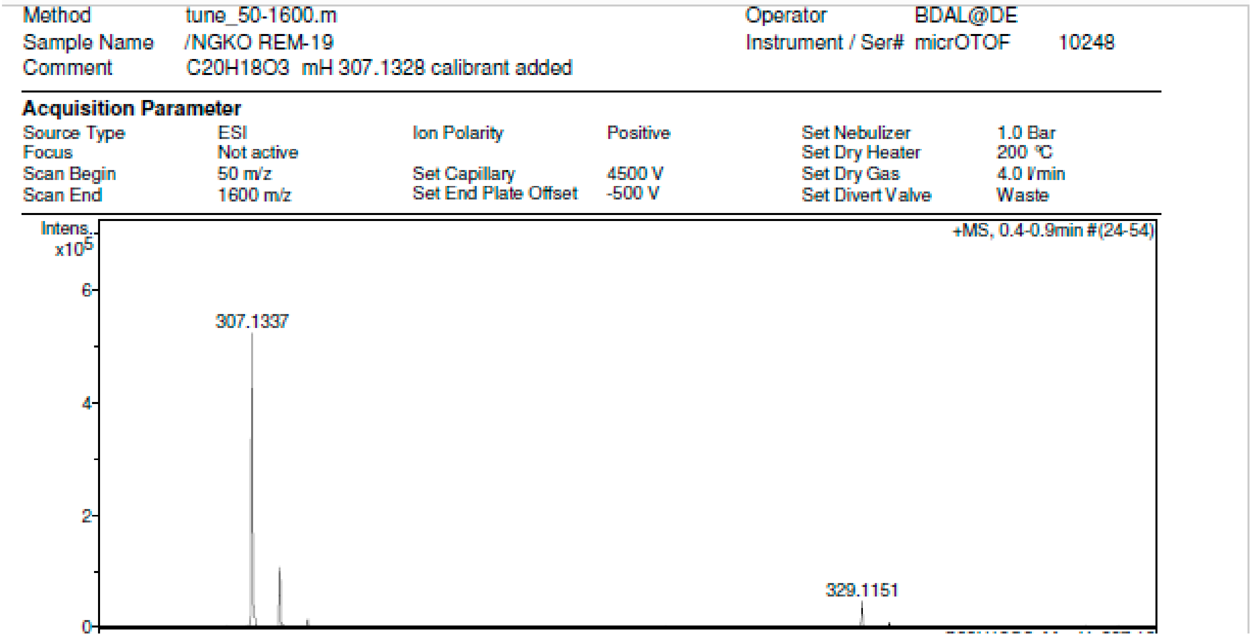

Figure S6. HRMS spectrum of compound **1b**.

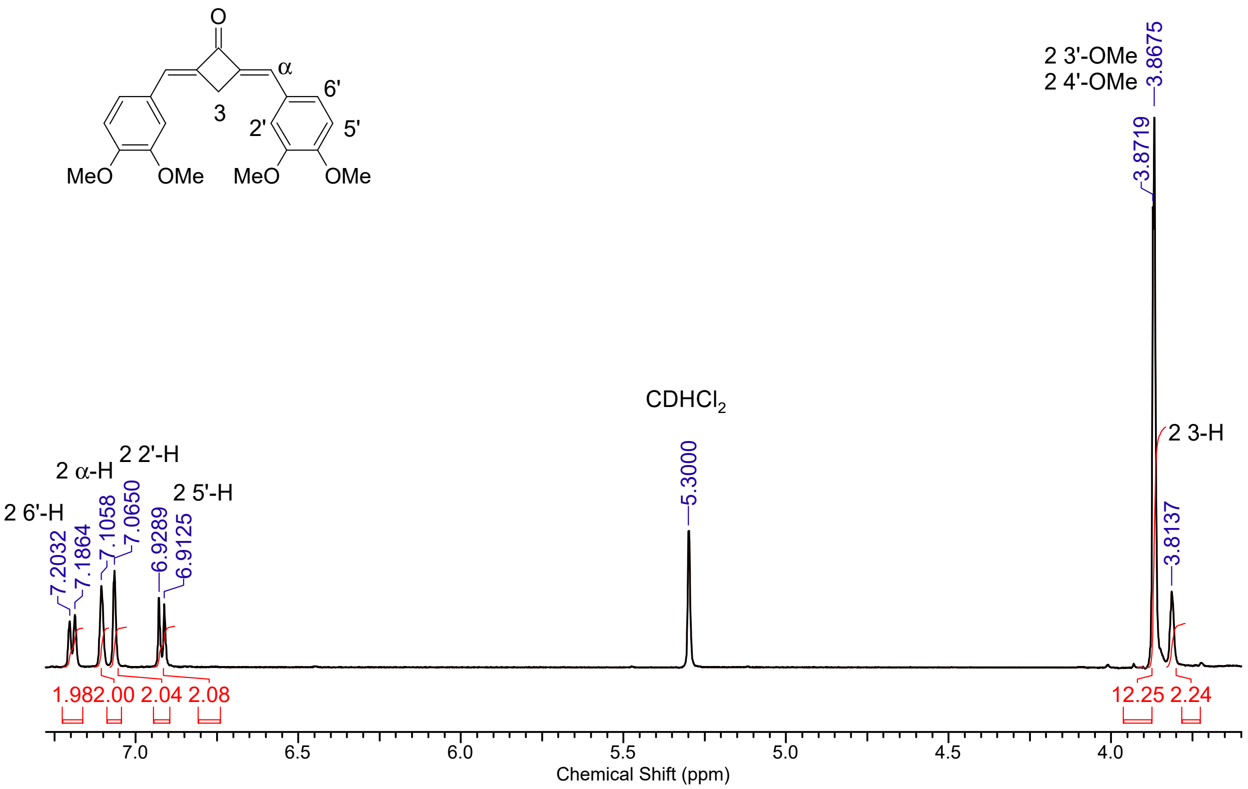

Figure S7. ^{1}H NMR spectrum of compound **1c** in CD_2Cl_2.

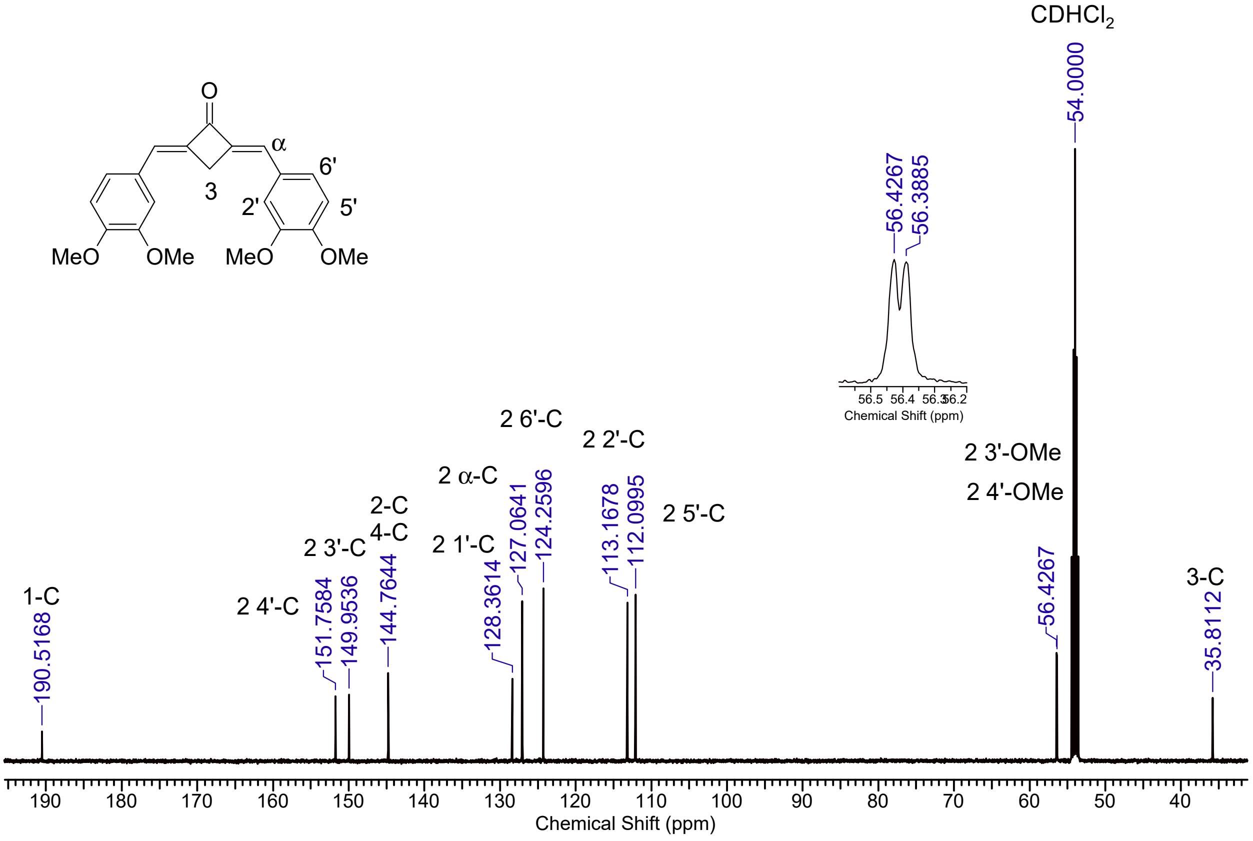

Figure S8. ^{13}C NMR spectrum of compound **1c** in CD_2Cl_2.

NOESY Spectrum

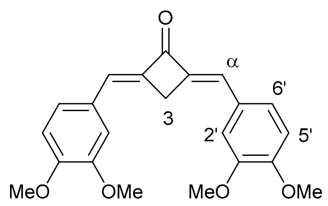

1c

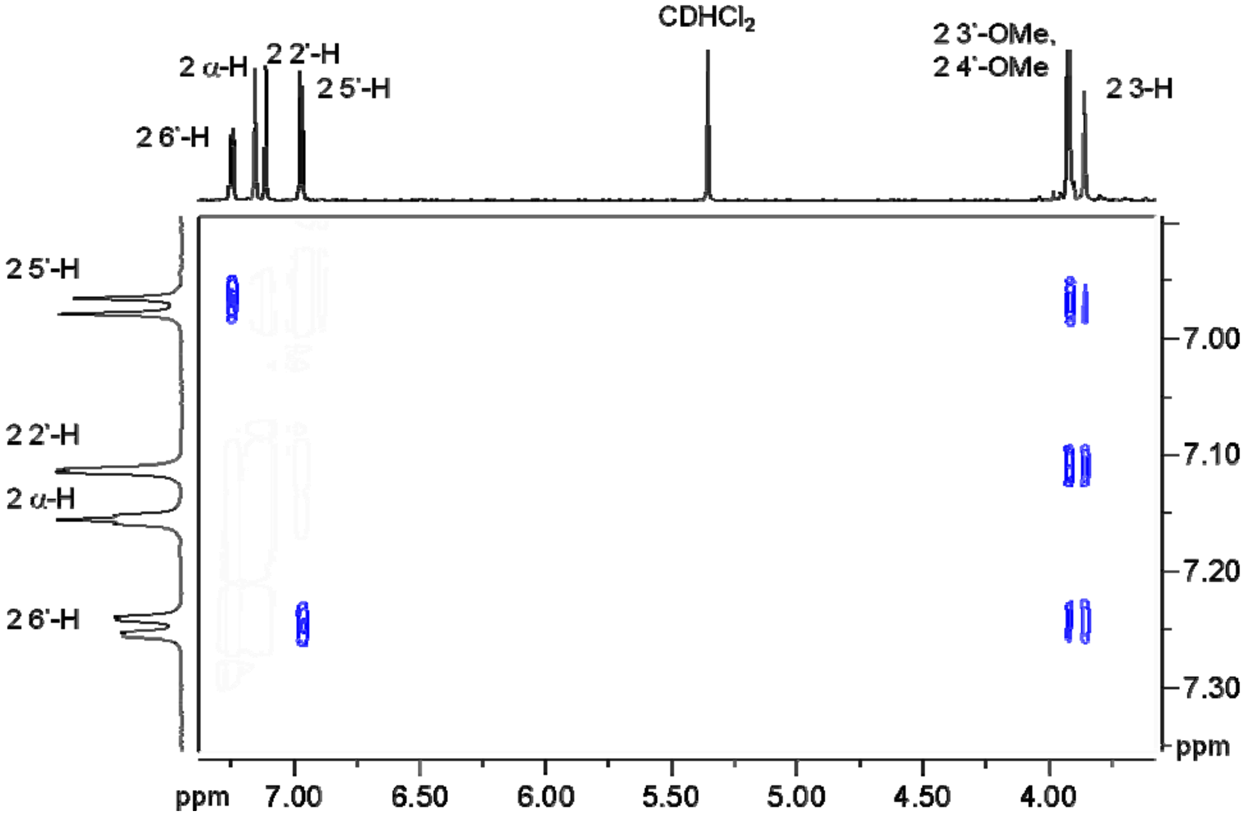

Figure S9. Fragment of the aromatic region of the NOESY (296 K, 600 MHz) spectrum of compound **1c** in CD_2Cl_2.

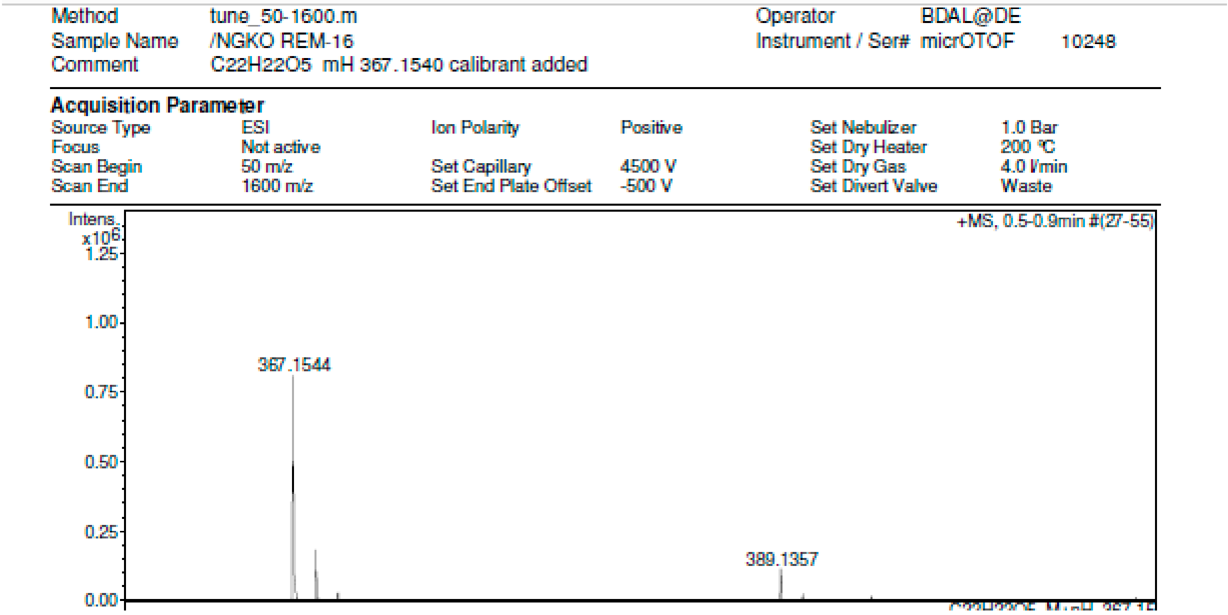

Figure S10. HRMS spectrum of compound **1c**.

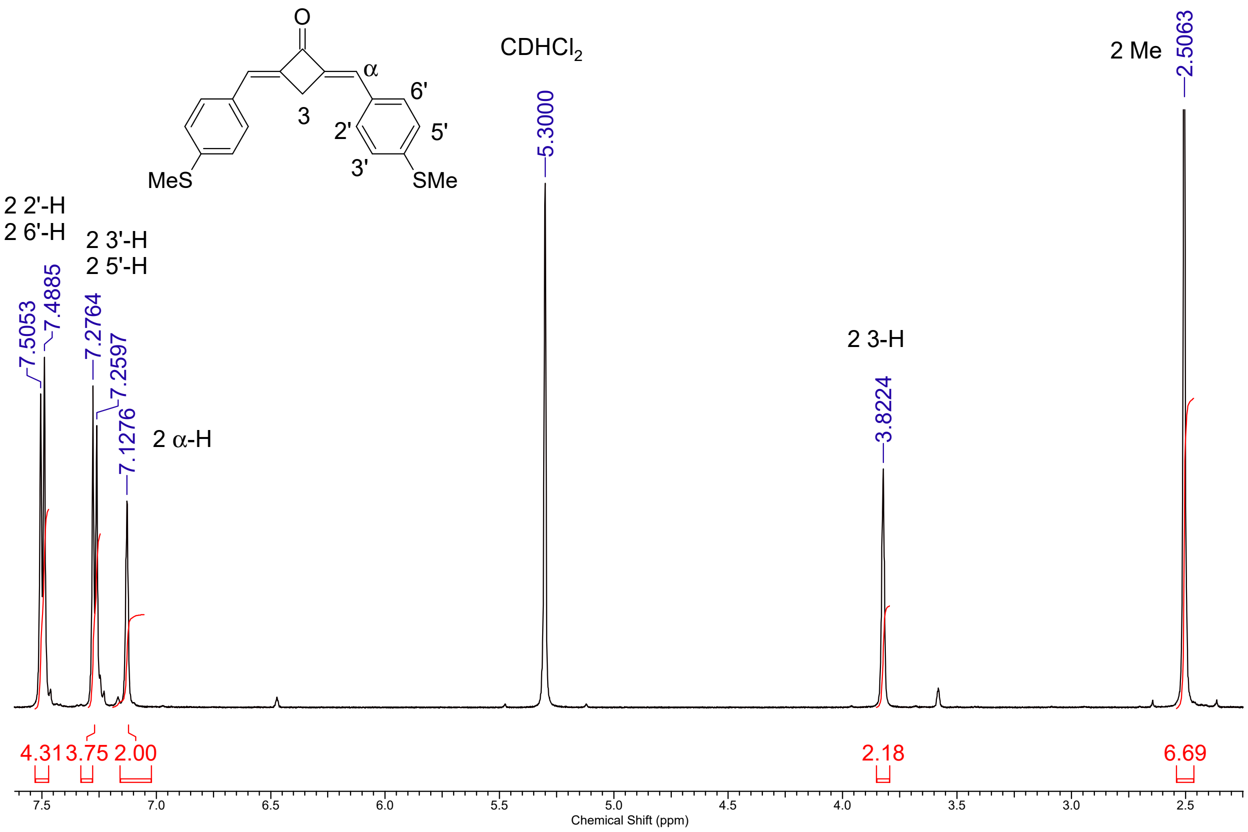

Figure S11. ^{1}H NMR spectrum of compound **1d** in CD_2Cl_2.

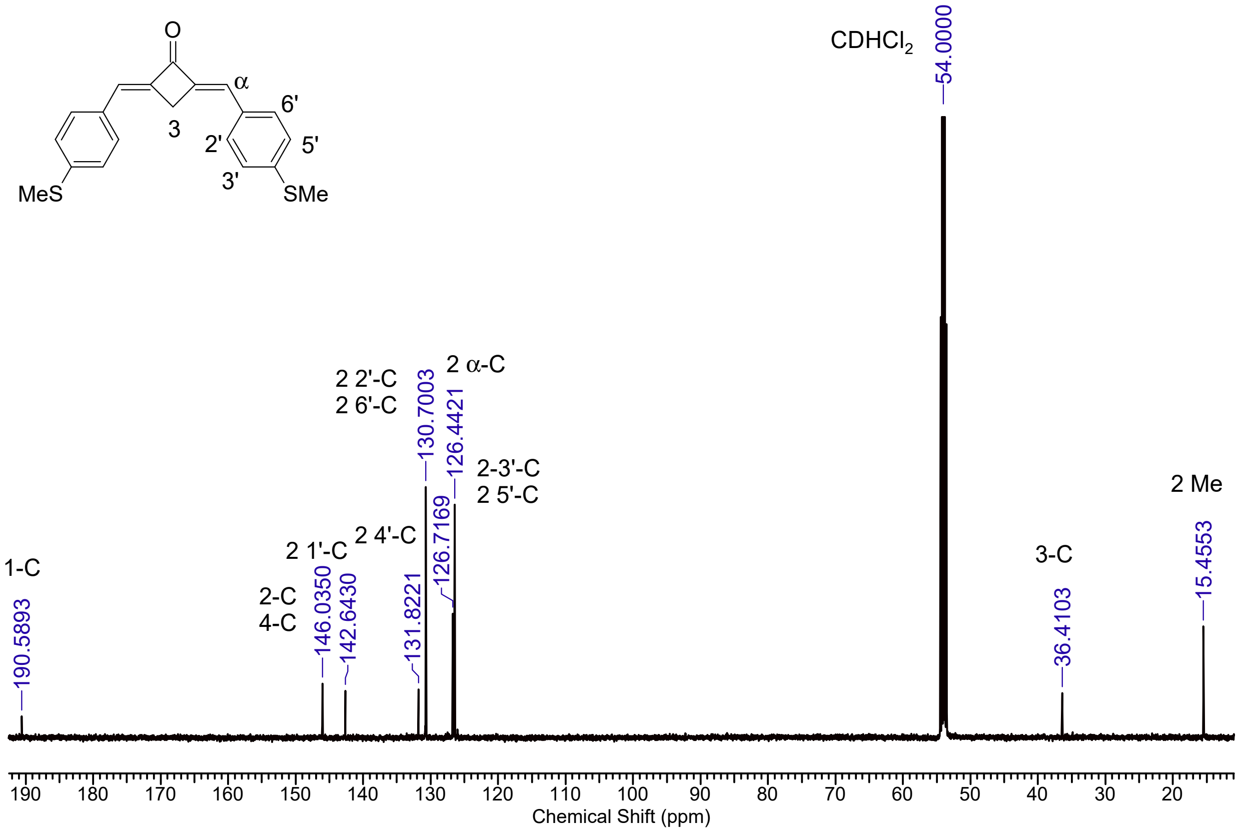

Figure S12. ^{13}C NMR spectrum of compound **1d** in CD_2Cl_2.

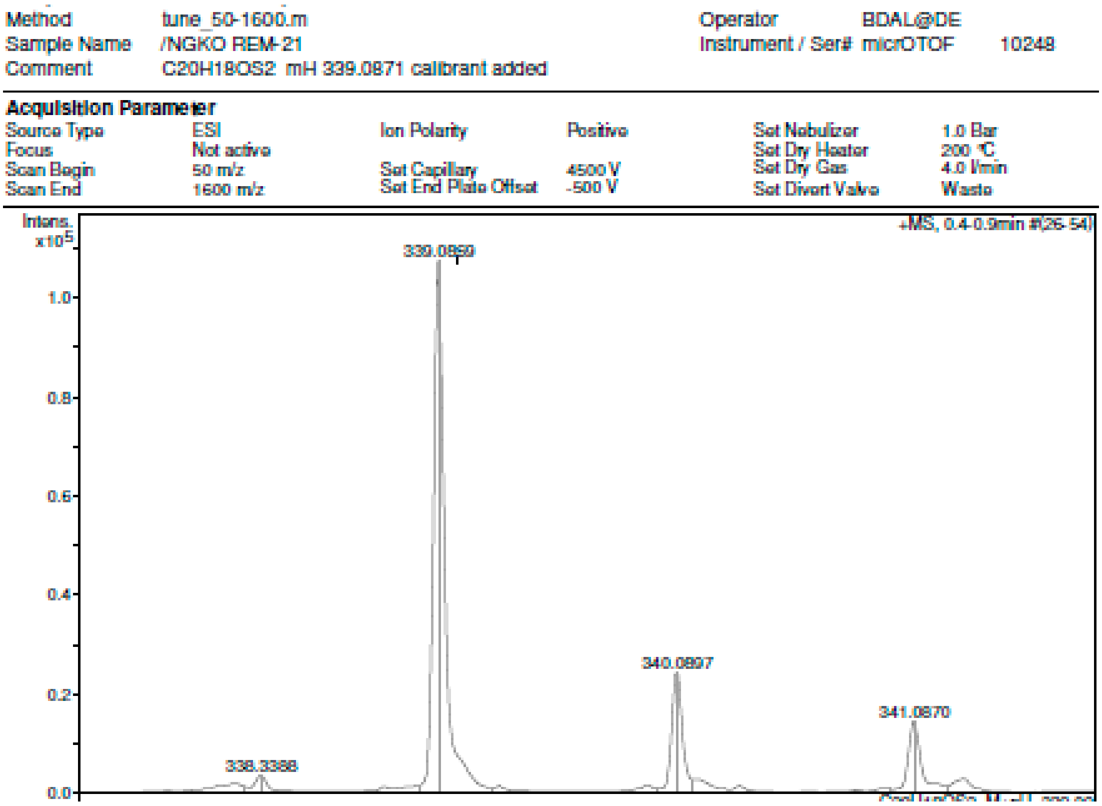

Figure S13. HRMS spectrum of compound **1d**.

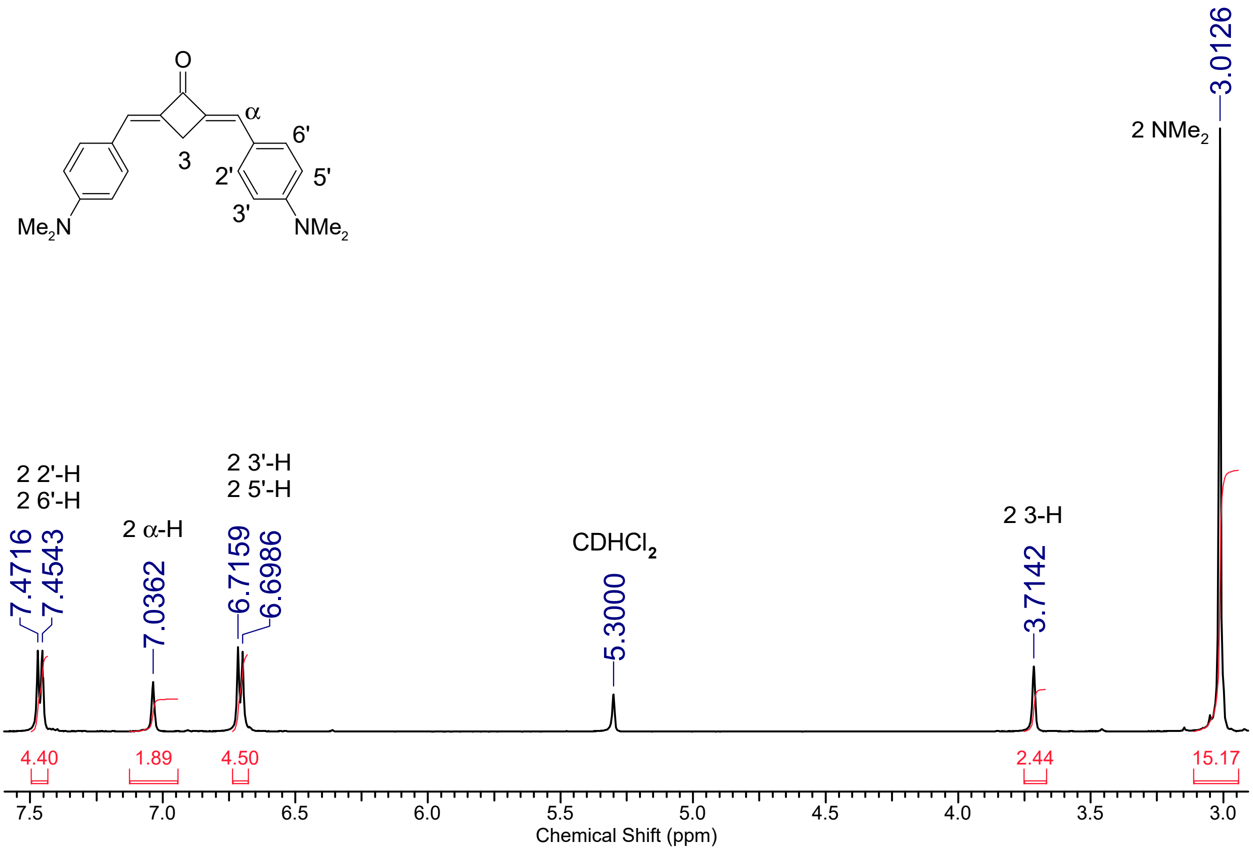

Figure S14. ^{1}H NMR spectrum of compound **1e** in CD_2Cl_2.

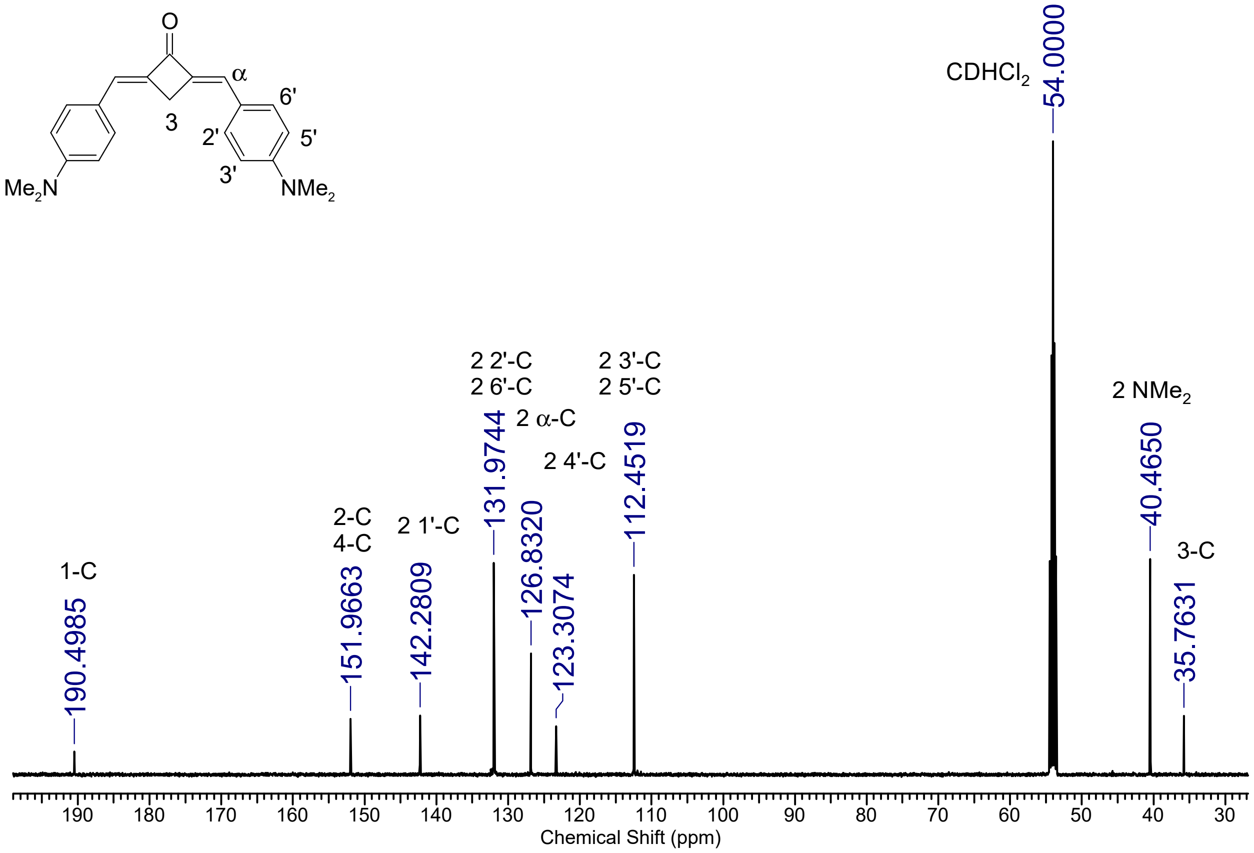

Figure S15. ^{13}C NMR spectrum of compound **1e** in CD_2Cl_2.

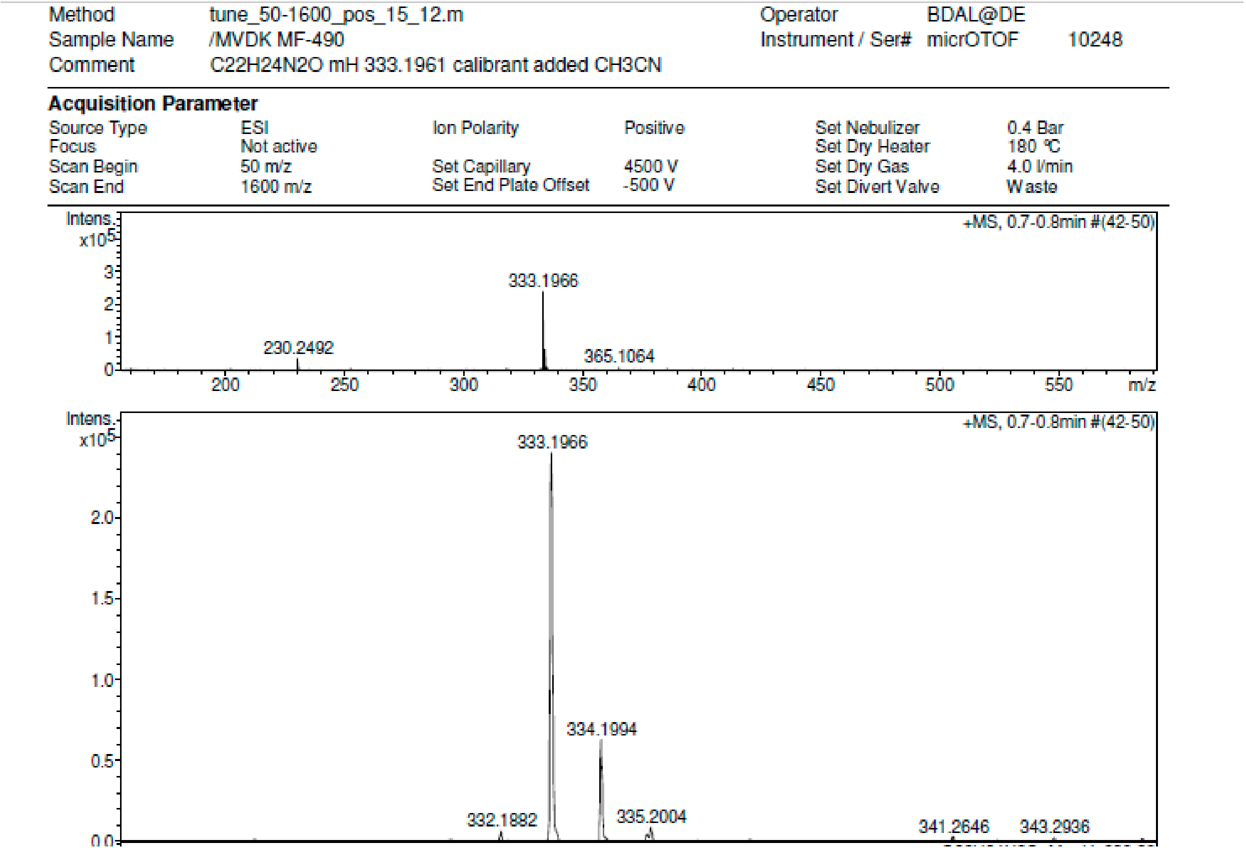

Figure S16. HRMS spectrum of compound **1e**.

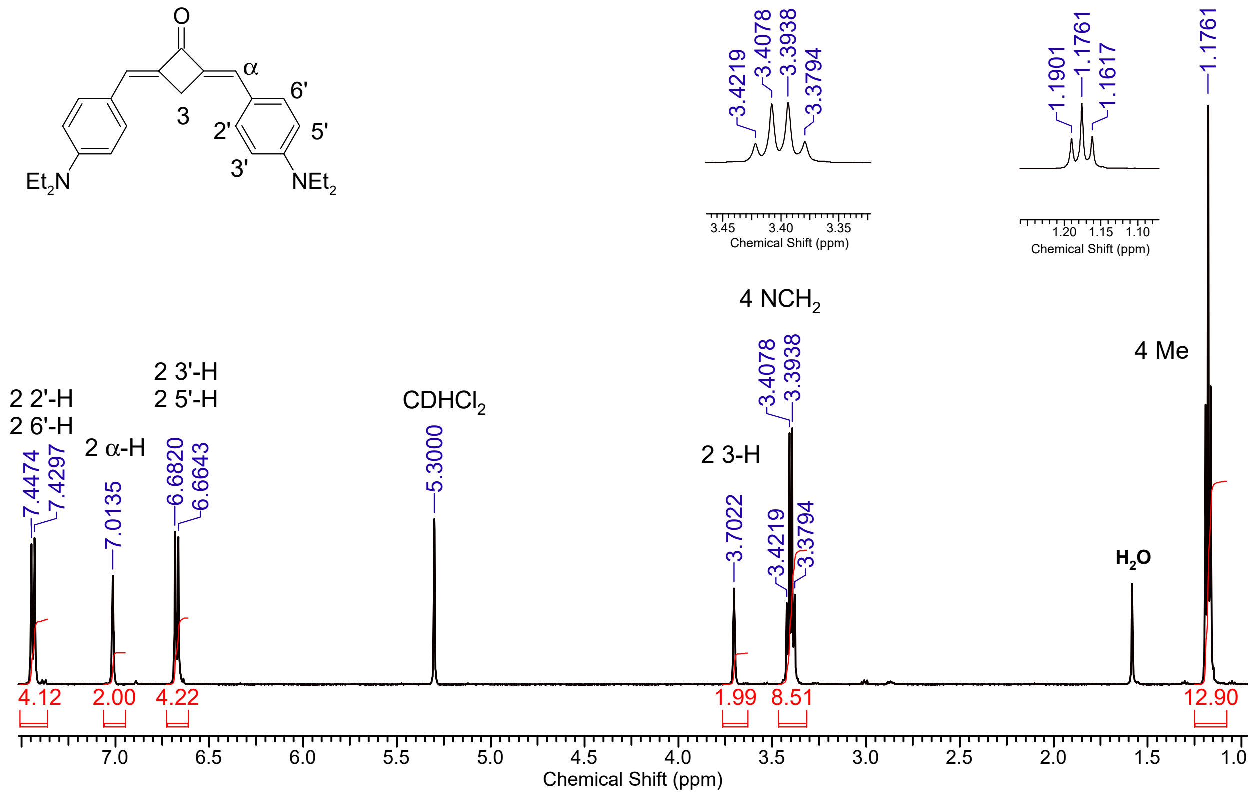

Figure S17. ^{1}H NMR spectrum of compound **1f** in CD_2Cl_2.

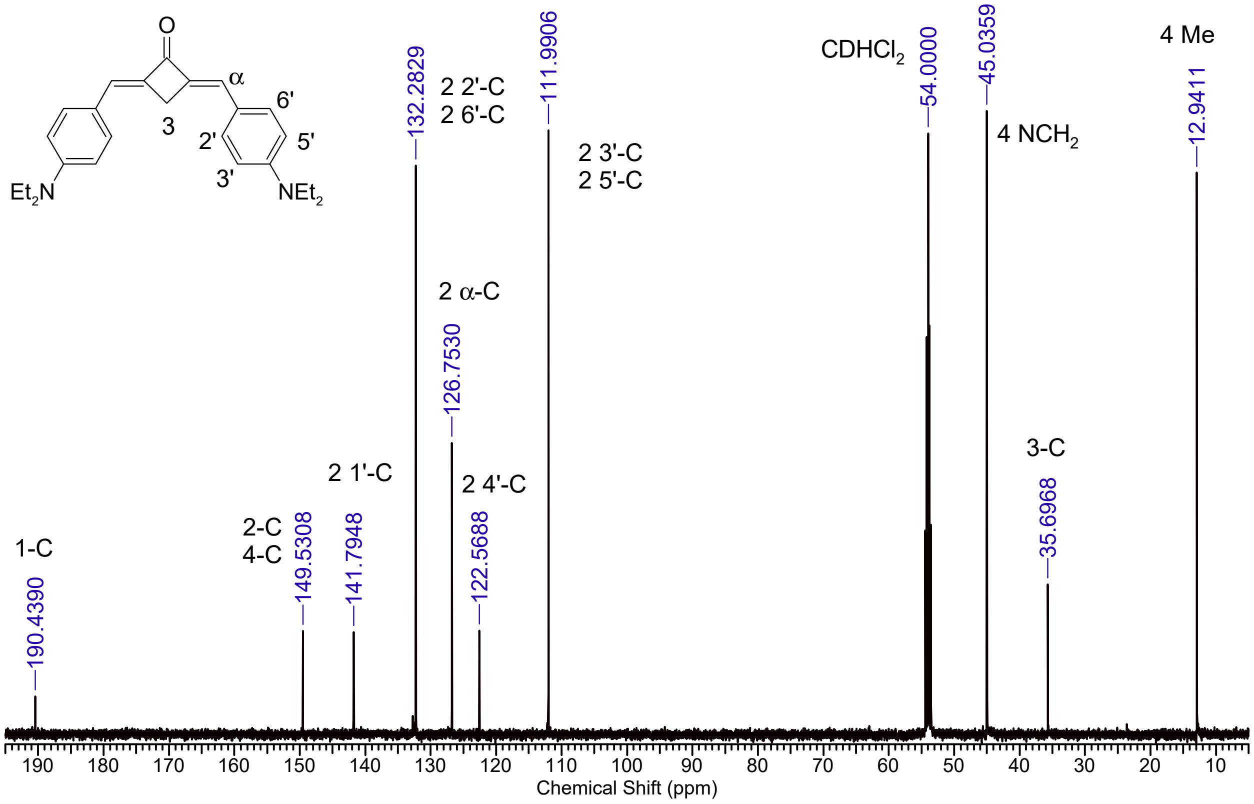

Figure S18. ^{13}C NMR spectrum of compound **1f** in CD_2Cl_2.

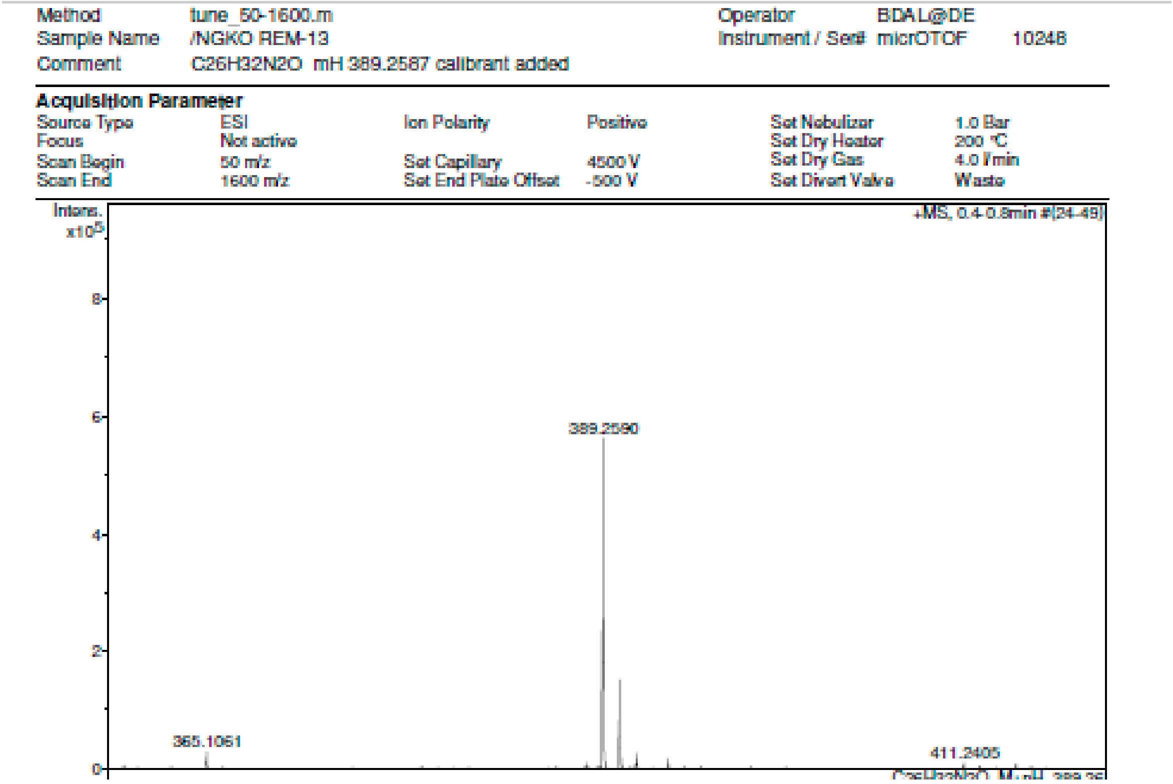

Figure S19. HRMS spectrum of compound **1f**.

III Fluorescence spectra of compounds 1c-f (Figure S20)

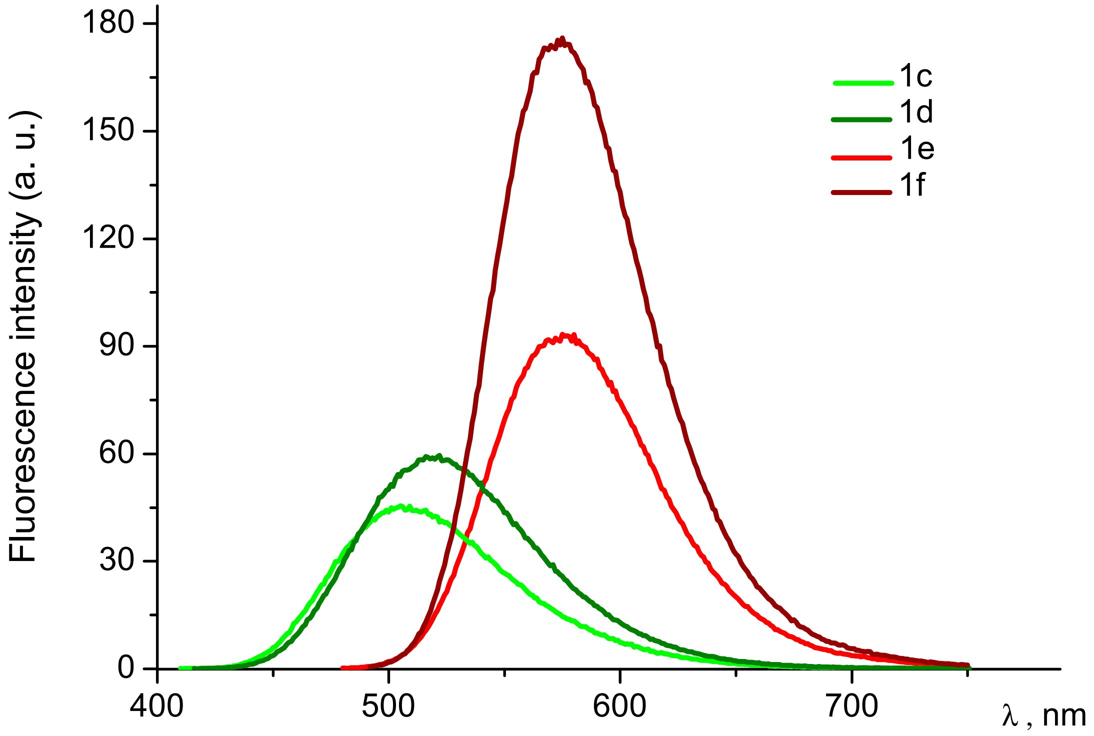

Figure S20. Fluorescence spectra of dienones **1c-f** ($C = 0.5{\times}10^{-6}$ M) in MeCN.

IV Quantum chemical calculations, orbitals involved in the first electron transition of compounds 1b-f (Figures S21-S25)

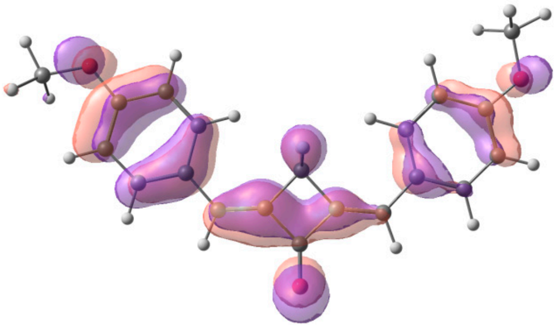

π orbital (HOMO-1) of compound **1b**

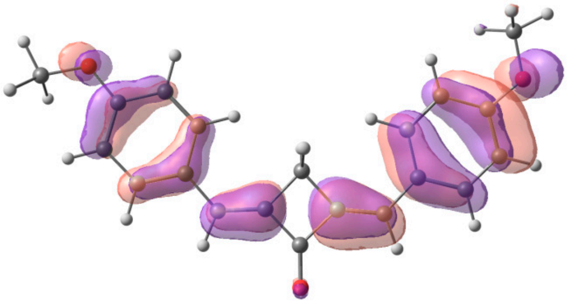

π orbital (HOMO) of compound **1b**

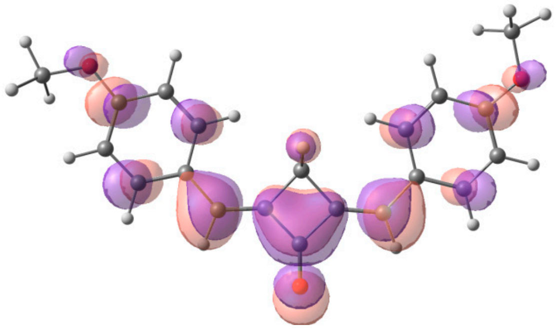

π^* orbital (LUMO) of compound **1b**

Figure S21. Orbitals involved in the first electron transition of compound **1b**.

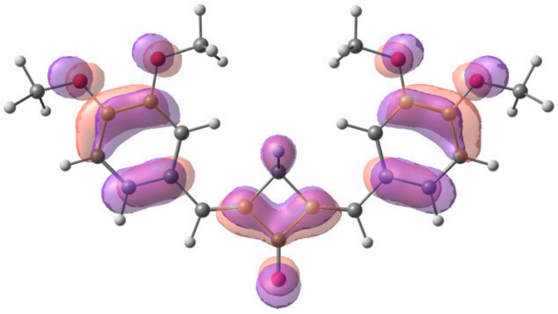

π orbital (HOMO-1) of compound **1c**

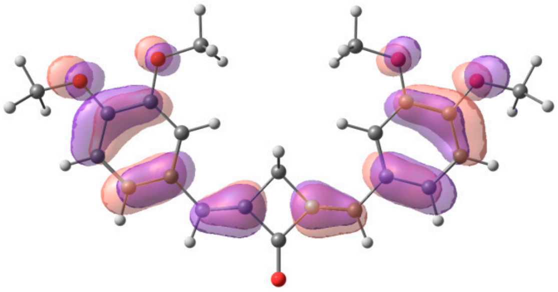

π orbital (HOMO) of compound **1c**

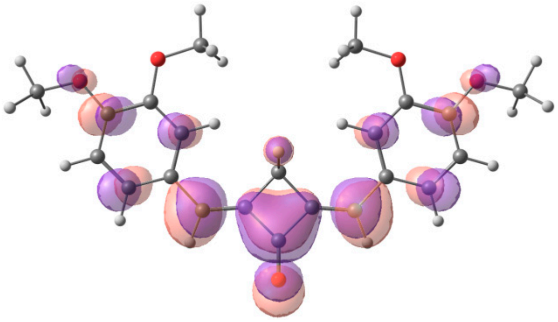

π* orbital (LUMO) of compound **1c**

Figure S22. Orbitals involved in the first electron transition of compound **1c**.

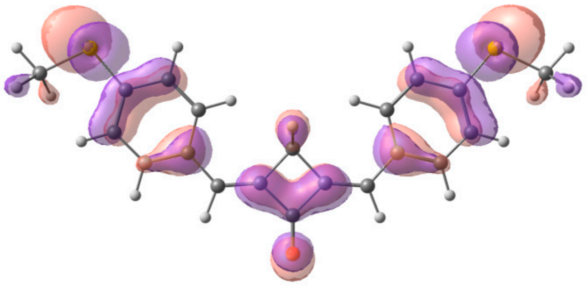

π orbital (HOMO-1) of compound **1d**

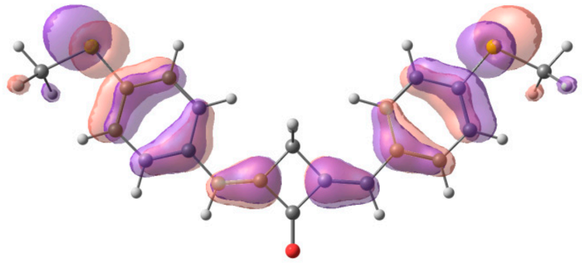

π orbital (HOMO) of compound **1d**

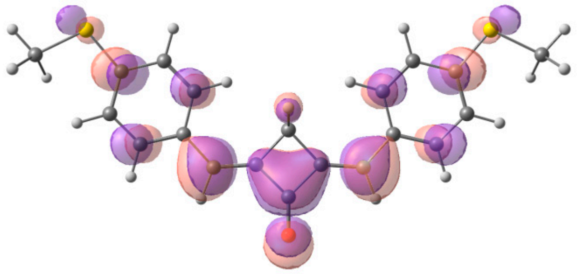

π^* orbital (LUMO) of compound **1d**

Figure S23. Orbitals involved in the first electron transition of compound **1d**.

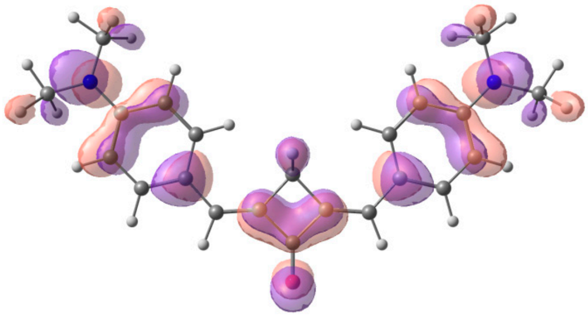

π orbital (HOMO-1) of compound **1e**

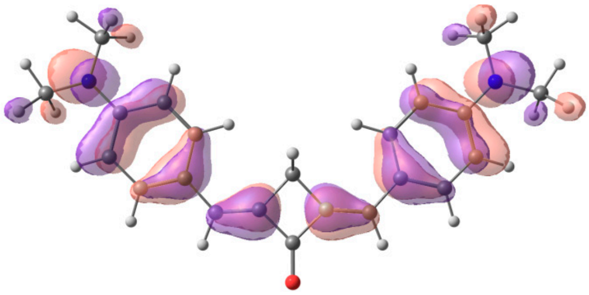

π orbital (HOMO) of compound **1e**

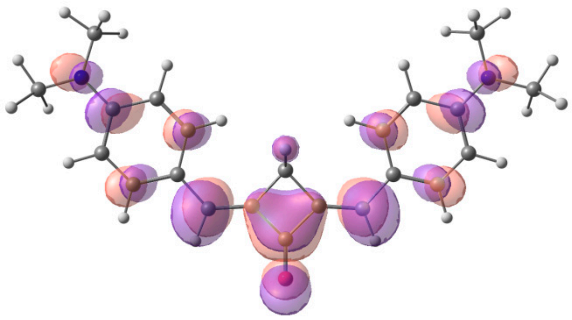

π^* orbital (LUMO) of compound **1e**

Figure S24. Orbitals involved in the first electron transition of compound **1e**.

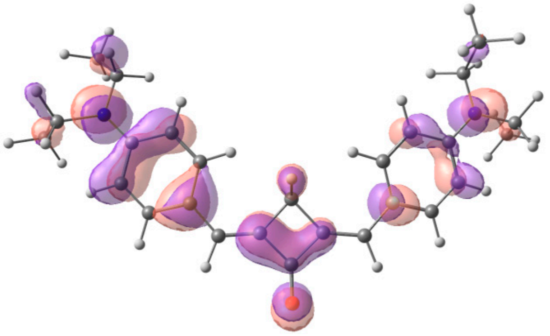

π orbital (HOMO-1) of compound **1f**

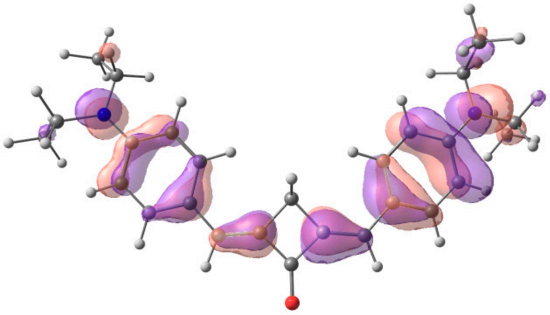

π orbital (HOMO) of compound **1f**

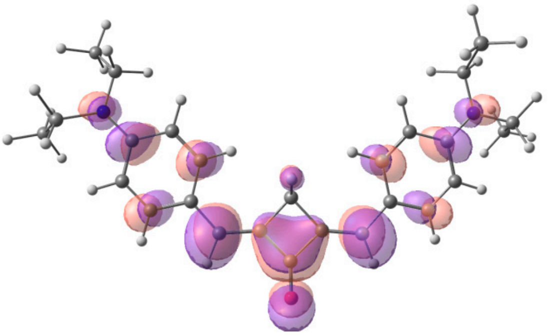

π^* orbital (LUMO) of compound **1f**

Figure S25. Orbitals involved in the first electron transition of compound **1f**.

V Quantum chemical calculations, potential energy profiles of the ground S0 and lowest excited states of compounds 1a-f (Figures S26-S32)

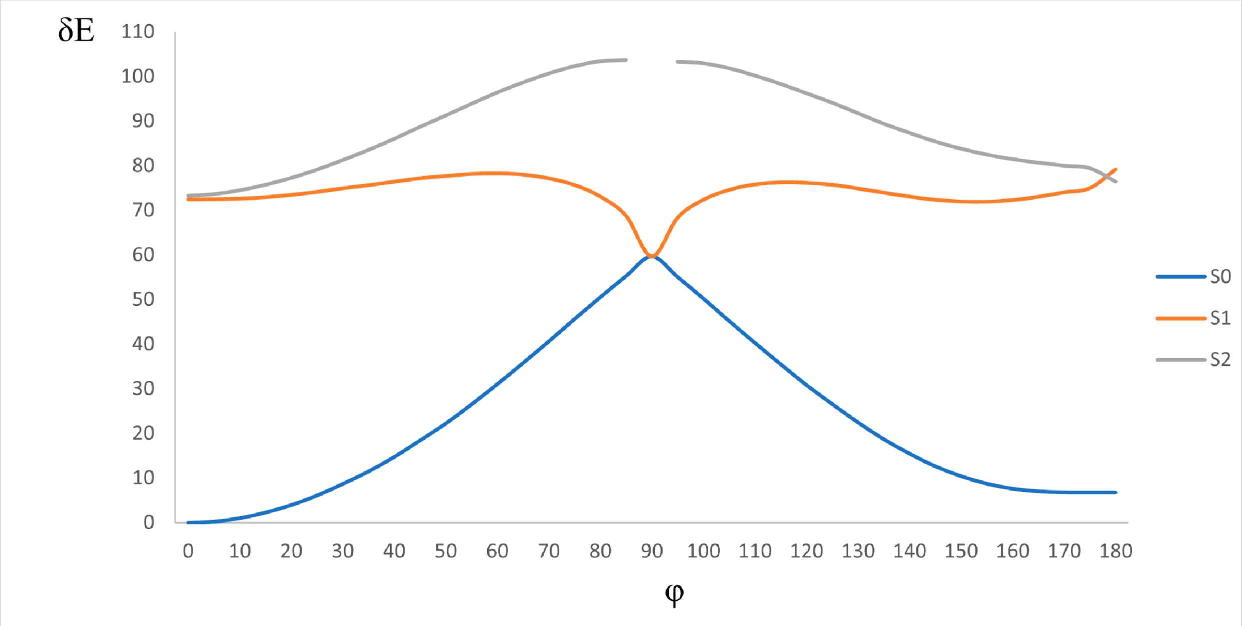

Figure S26. Potential energy profiles of compound **1a**.

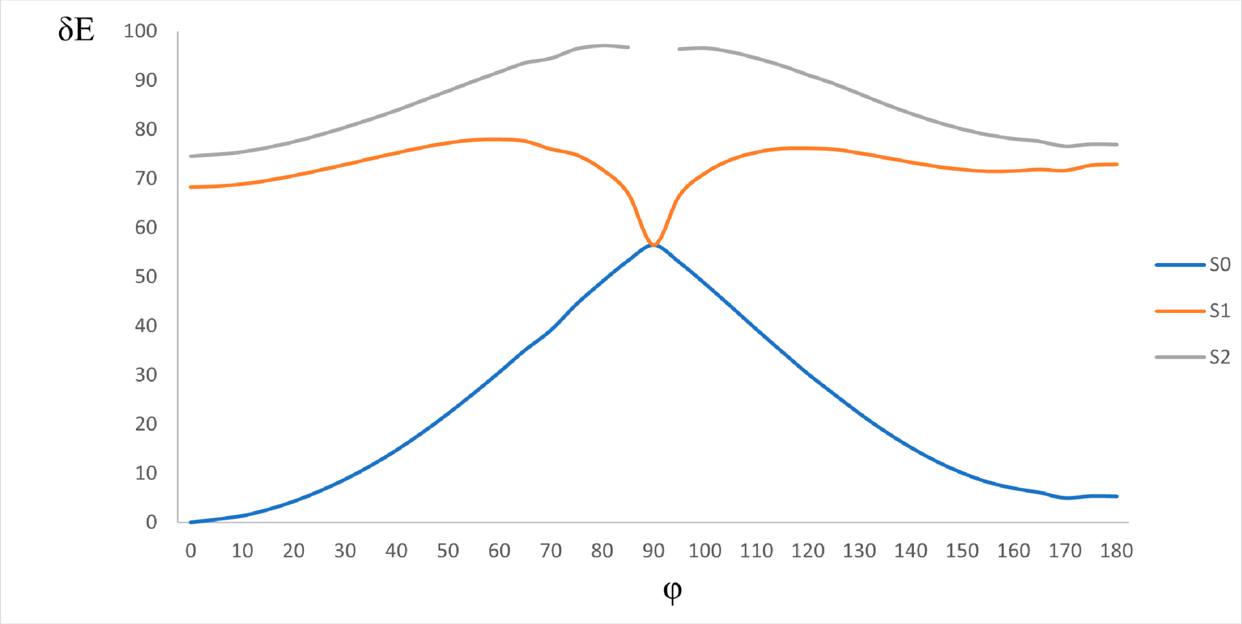

Figure S27. Potential energy profiles of compound **1b**.

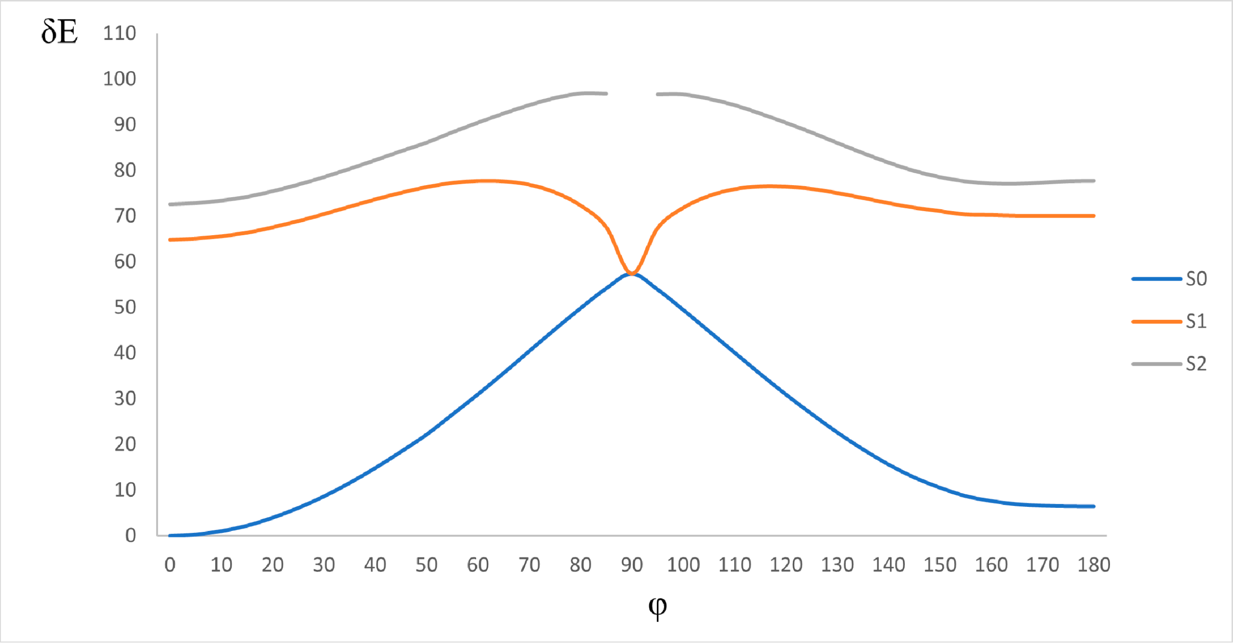

Figure S28. Potential energy profiles of compound **1c**.

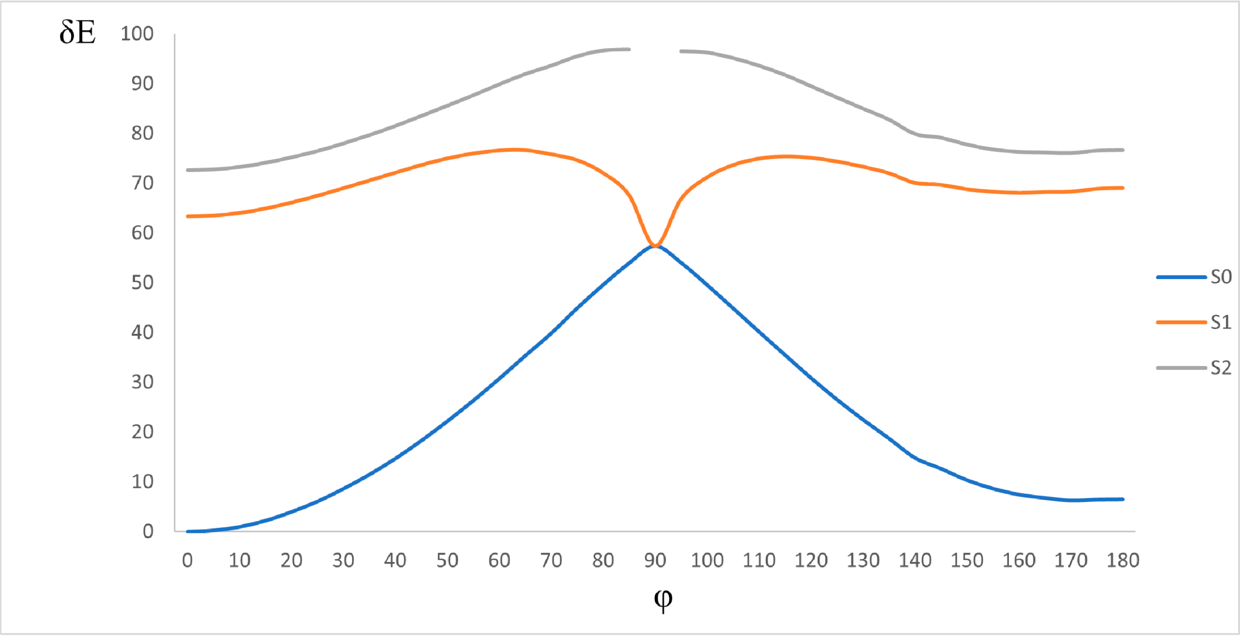

Figure S29. Potential energy profiles of compound **1d**.

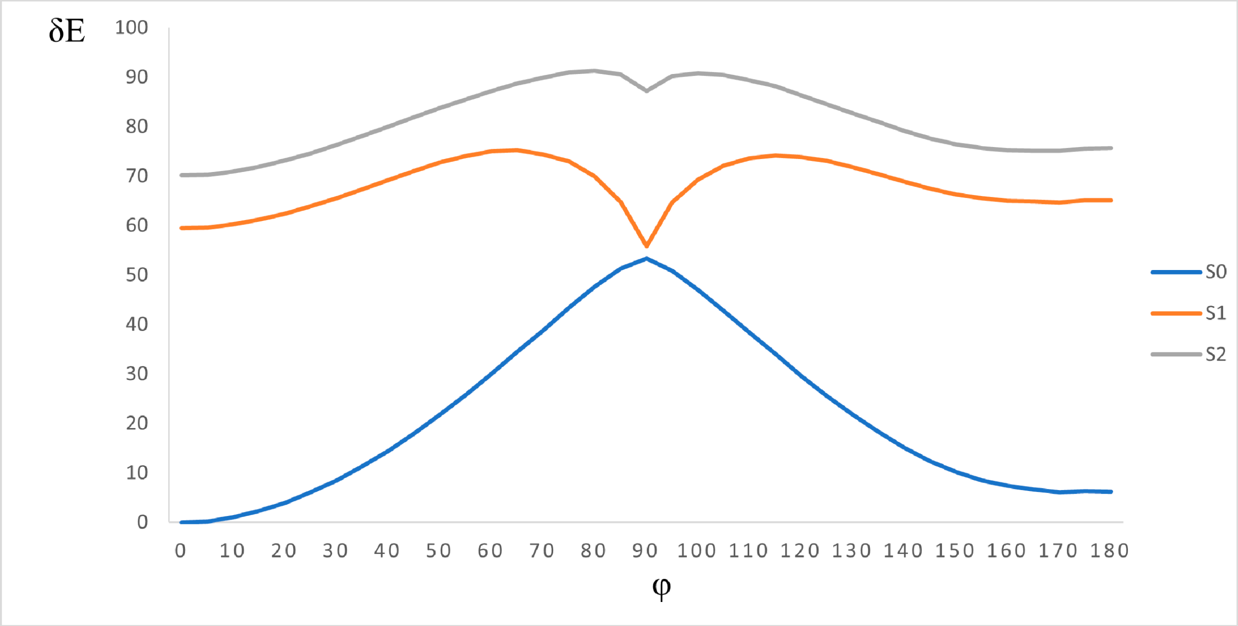

Figure S30. Potential energy profiles of compound **1e**.

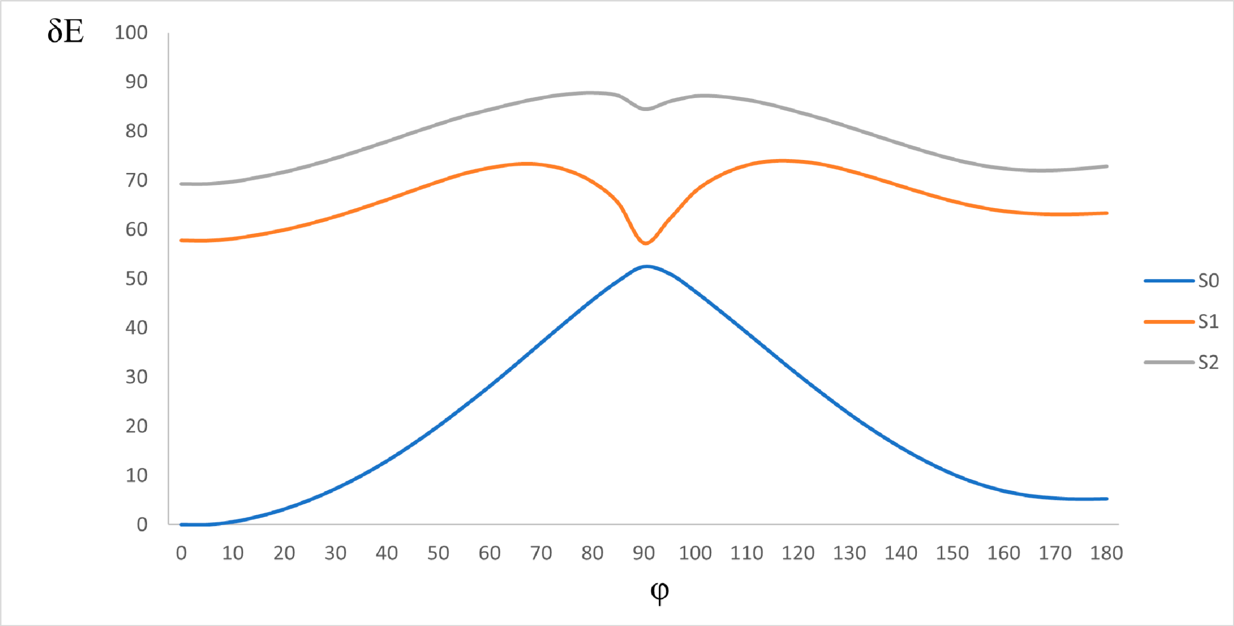

Figure S31. Potential energy profiles of compound **1f**.

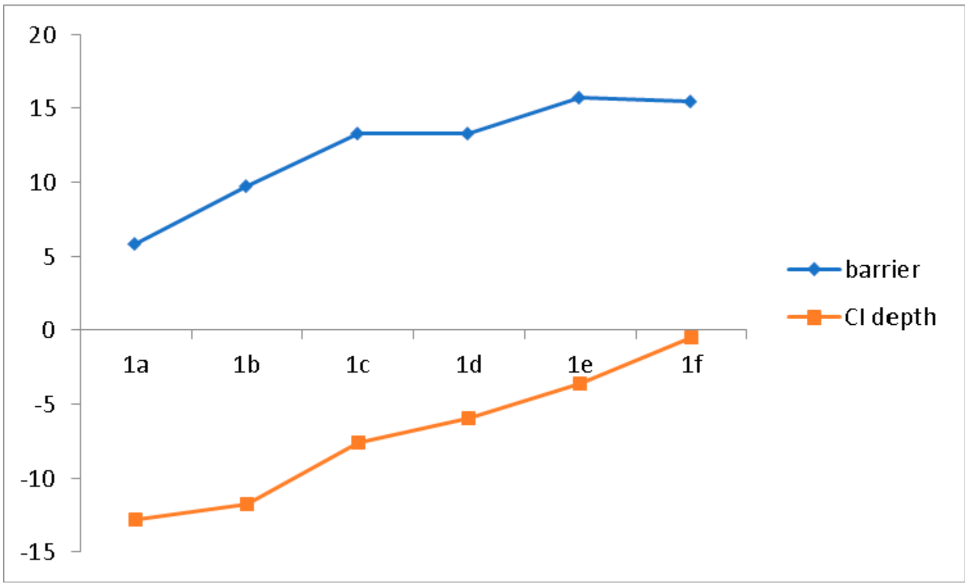

Figure S32. *E*-*Z* isomerization barrier height and CI depth in the series.

VI Correlations between the calculated frontier orbital energies, ionization potential and electron affinities, and experimental oxidation and reduction potentials (Figures S33-S35)

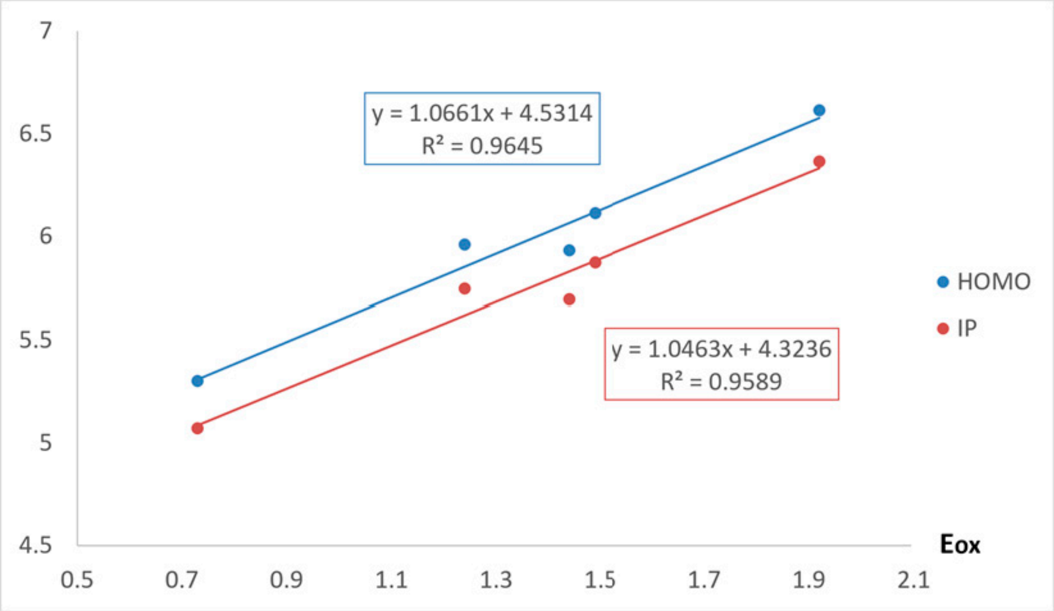

Figure S33. Correlations of HOMO, HOMO-1 energy, and calculated ionization potential with E_{ox}. Color of the frame with regression equation and correlation coefficient corresponds to the color of the trend line.

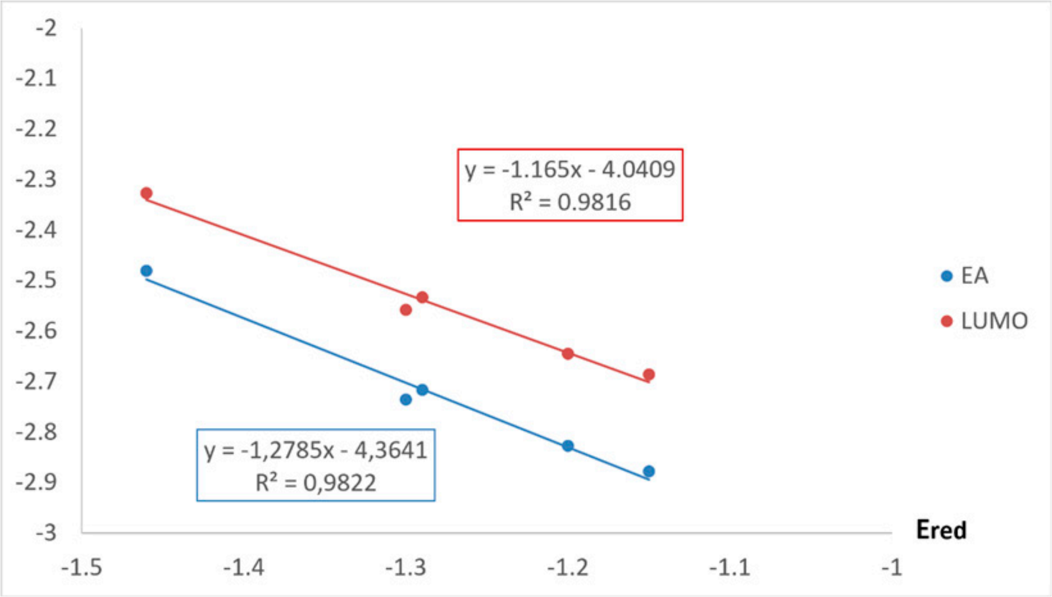

Figure S34. Correlations of LUMO energy and calculated electron affinity with E_{red}. Color of the frame with regression equation and correlation coefficient corresponds to the color of the trend line.

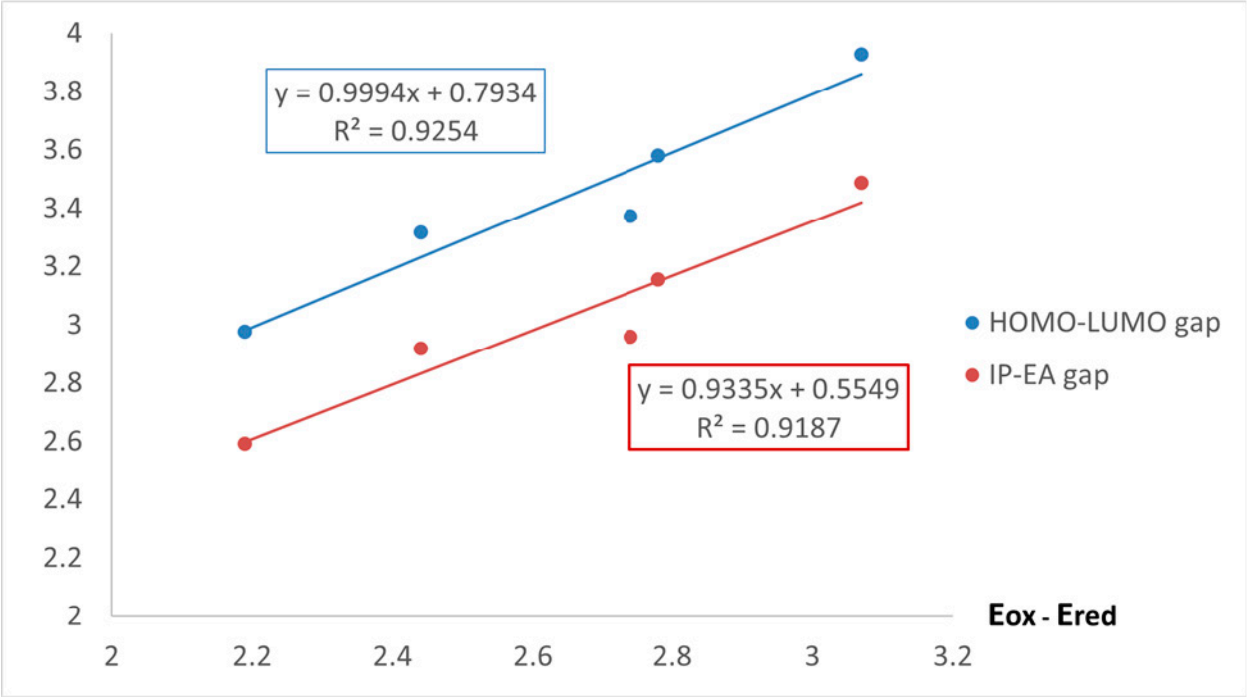

Figure S35. Correlations of the HOMO-LUMO gap and IP–EA difference with E_{ox}–E_{red}. Color of the frame with regression equation and correlation coefficient corresponds to the color of the trend line.
